# Assessment of global hydro-social indicators in water resources management

**DOI:** 10.1038/s41598-021-96776-9

**Published:** 2021-08-31

**Authors:** Omid Bozorg-Haddad, Sahar Baghban, Hugo A. Loáiciga

**Affiliations:** 1 Department of Irrigation and Reclamation Engineering, Faculty of Agriculture Engineering and Technology, College of Agriculture and Natural Resources, University of Tehran, Karaj, Alborz Iran; 2 Department of Geography, University of California, Santa Barbara, CA 93016-4060 USA

**Keywords:** Climate sciences, Ecology, Environmental sciences, Environmental social sciences, Hydrology, Natural hazards, Solid Earth sciences, Engineering, Mathematics and computing

## Abstract

Water is a vital element that plays a central role in human life. This study assesses the status of indicators based on water resources availability relying on hydro-social analysis. The assessment involves countries exhibiting decreasing trends in per capita renewable water during 2005–2017. Africa, America, Asia, Europe, and Oceania encompass respectively 48, 35, 43, 20, and 5 countries with distinct climatic conditions. Four hydro-social indicators associated with rural society, urban society, technology and communication, and knowledge were estimated with soft-computing methods [i.e., artificial neural networks, adaptive neuro-fuzzy inference system, and gene expression programming (GEP)] for the world’s continents. The GEP model’s performance was the best among the computing methods in estimating hydro-social indicators for all the world’s continents based on statistical criteria [correlation coefficient (R), root mean square error (RMSE), and mean absolute error]. The values of RMSE for GEP models for the ratio of rural to urban population (PRUP), population density, number of internet users and education index parameters equaled (0.084, 0.029, 0.178, 0.135), (0.197, 0.056, 0.152, 0.163), (0.151, 0.036, 0.123, 0.210), (0.182, 0.039, 0.148, 0.204) and (0.141, 0.030, 0.226, 0.082) for Africa, America, Asia, Europe and Oceania, respectively. Scalable equations for hydro-social indicators are developed with applicability at variable spatial and temporal scales worldwide. This paper’s results show the patterns of association between social parameters and water resources vary across continents. This study’s findings contribute to improving water-resources planning and management considering hydro-social indicators.

## Introduction

Water resources shortages are caused by climatic variability and change, population growth, and mismanagement posing challenges to meeting the water requirements in many countries^1,2^. Water resources management involves hydraulic and hydrologic issues and must consider social and economic conditions. Early hydro-social research of water systems relied heavily on geographic assessments and introduced methods for understanding the feedbacks between water and human systems^3^. Hydro-social studies are based on recognizing the close interactions between human systems and water, the social and cultural meanings of water, and how they relate to water systems and water management options^4^. The hydro-social cycle, focusing on the feedback systems between human and water interactions, recognizes the human impact on the hydrological cycle as part of the dialectical development of water systems and social systems^5^.

There are several definitions of water scarcity and different interpretations of its meaning. At the basic level water scarcity is governed by its quantity and distribution and by natural and human factors^2^. Human populations are affected by water stresses and per capita shortages of renewable water. Water scarcity is generally considered as a global challenge for humanity^6^. Human actions have caused a diverse set of water sustainability challenges that must be addressed by new approaches to water management^7^. Research has shown that water scarcity is the result of physical water scarcity and the result of complex interactions between water resources and social phenomena^6^. In addition to the social and economic crises of water, it has been emphasized that the water problem is not simply about scarcity but a crisis of water management. Today's goal in managing water systems is to define new interdisciplinary solutions. Hydro-social science studies the interactions between human factors, water flows, hydraulic technologies, biophysical elements, socio-economic structures, and cultural-political institutions in the management of water systems^8^. This science evaluates water resource systems considering human influences such as withdrawals, impoundments, and other human-induced changes in hydrological systems^9^. This means that water shortages and the existence of adverse trends in hydrological systems also affect human interests, and, ultimately, social wellbeing^10^. Therefore, modeling and predicting these relationships is useful for understanding the hydro-social interactions for more efficient management of water resources and improved societal assessments.

Hydro-social indicators are useful measures of the relative magnitude of phenomena^11^, and they support decision making in water resources management. These indicators are evaluated to assess the effectiveness of current policies and future management activities and investment decisions based on factors such as the definition or perception of water among stakeholders, the primary mechanisms of water perception by stakeholder groups (e.g., social, historical, economic, political, spiritual, etc.), the feedbacks between water and society that create a "water community", and the consequences of hydro-social interactions between stakeholders and their Water^12,13^. Understanding hydro-social relationships raises awareness about best practices for water use^14^.

The importance and indivisible relationship between social parameters and comprehensive water resources management is nowadays well understood. Understanding the feedbacks between social parameters and per capita renewable water and its modeling yields predictions about countries' future social evolution. Soft computing methods and artificial-intelligence-based methods such as Artificial Neural Network (ANN), adaptive neuro-fuzzy inference system (ANFIS), gene expression programming (GEP), Multivariate Adaptive Regression Splines (MARS), the M5 Tree model, Support Vector Machines (SVM), Random Forests (RF), and Multi Linear Regression (MLR) methods have been successfully employed in water quality and quantity modeling. These data-based prediction methods have been used to predict various phenomena in different fields, including water resources management.

Hydro-social science evaluates the relationship between water science and social science and discovers mathematical functions that predict phenomena in these two sciences. Hydro-social research stems mainly from the convergence of political ecology and technology studies^3^. Ross et al.^3^ argued that most studies on water resources systems in the context of hydro-social issues are related to irrigation, water scarcity, dams, groundwater, desalination, glaciers, sanitation, and mining. Carey et al.^15^ introduced social sciences in the hydrological modeling of glacial basins. They studied glacier melting caused by climate change in the Santa River Basin, Peru, employing five variables: political agendas and economic development, governance (laws and institutions), technology and engineering, land and resource use, societal responses. The latter authors presented a hydrological modeling tool in hydro-social science to understand the impact of climate change on glacier shrinkage that affects the human population. Chen et al.^16^, applied ordinary least squares (OLS) and geographic weight regression (GWR) models to identify the effect of land use and population density indices on surface water quality in wet and dry seasons in the Wen-Rui Tong River basin in eastern China. Their results revealed that the impact of these indicators varies with the spatial and seasonal scales. Suburban and rural areas were identified with urban land as the primary influencing factor concerning pollutants during the wet season, while agricultural land was identified as a more prevalent influencing factor during the dry season. Ženko et al.^6^ investigated the effect of water shortage on water users' mental health based on gender and age group in Iran's Urmia Lake basin, and determined that water scarcity adversely affect the economy, social relations, and people's health. At the same time, all these factors threaten the mental health of water users. The latter authors evaluated hydro-social factors and showed that water problems lead to biophysical, financial, and social changes that impact the health of water users due to chronic psychological stress, social isolation, intra-community conflicts, despair, hopelessness, depression, and anxiety. Shrestha et al.^17^ analyzed competition and conflict over water scarcity in the Kathmandu Valley, Nepal, and showed human distress due water insecurity and the inability to integrate political, social, and economic factors to allow access to water services and institutions. Devkota et al.^18^ applied hydrological analysis and flood modeling in the West Rapti River basin by a community survey of 240 households based on public perceptions. They examined flood adaptation strategies that had already occurred or are likely to occur in the future and applied a hybrid hydro-social approach to demonstrate the importance of flood plans to raise local flood awareness. Weigleb et al.^19^ evaluated the path from MDG to SDG to achieve the Sixth Sustainable Development Goal (SDG 6). The key factors in this respect are the management of problem sources and not their effects, increasing integration of issues and sectors, inclusion of environmental goals, more flexible management approaches, participation and collaborative decision-making, more attention to managing human behavior through "soft measures", open and shared information systems, and incorporation of learning cycles. They considered the "hydro-social cycle" a concept to connect society and the vital water element. Bui et al.^20^ assessed groundwater resources' social sustainability in Hanoi, Vietnam, concerning three main groundwater characteristics (quantity, quality, and management). The sustainability indices, quantity, quality, and management of groundwater were estimated good, poor, and acceptable with the values 0.68, 0.27, and 0.52, respectively, which resulted in Hanoi being rated at an acceptable level with the value of 0.49 for the social sustainability assessment. Pande et al.^21^, exploiting the theory of metabolism, explored the relationship between birth rate and local water consumption by considering the virtual water content, virtual water trade, and agricultural production at the 7-continent scale. They investigated whether the average rate of human metabolism controls or is controlled by per capita water consumption, and reported that continents with relatively low birth rates, including North America, Europe, and Oceania, feature relatively high per capita water consumption, while developing regions exhibit an opposite pattern of association. Diaz et al.^22^ implemented the Driver-Pressure-State-Impact-Response (DPSIR) to explore the association between river ecosystems and the social system of the Biobío Basin in Chile. 65 indicators whose data spans over a period of 35 years were selected for assessing the DPSIR in the study area. The trend analysis results indicate a significant reduction in biodiversity, the deterioration of regulatory services and non-material goods for human well-being, while cultural services, direct and indirect pressures, and institutional responses increased. Forouzani et al.^2^ applied the Q method (a social sciences technique useful for discerning views, opinions, beliefs, attitudes) to identify farmers' and agricultural experts' understanding of agricultural water poverty and its causes in Marvdasht city, Iran. They surveyed the traits of agricultural water poverty with the Q method to identify four distinct types of farmers (management-adherents, adaptive-adherents, fatalists, and support seekers), and three types of agricultural specialists (farmer blamer pessimists, technocratic realists, and optimists). Li et al.^23^ examined the impact of various socio-economic activities on Lake Tai's water quality in China and demonstrated that severe ecological pressures from repeated and intense socio-economic activities can lead to the decline of the ecological functions of lakes and threaten aquatic organisms' health. Their results indicate a significant association between the average annual concentration of total nitrogen (TN), total phosphorous (TP), chemical oxygen demand (COD), biological oxygen demand (BOD), population, per gross domestic product (GDP), and sewage discharge. Several other studies have reported social-science and soft computing applications to water resources investigations^7,24–27^. On the other hand, the soft-computing methods literature is too vast to be reviewed in this paper; therefore, only a small set of references is herein highlighted that have applied soft computing methods.

Various researchers have examined the inclusion of social indicators in assessing water resources to understand how and which of the social parameters have the most significant impact on the water system; however, few have uncovered the patterns of association that govern hydro-social indicators quantitatively on a broad scale. It is essential to consider the rationality of the statistical association between social parameters and water resource parameters, and the level of interaction between these factors deserves further research.

This paper develops functions of worldwide application for water-resources factors within the context of hydro-social science. With respect to the current state-of-the-art in hydro-social science the innovations of this work are: (1) application of soft-computing methods (i.e., Artificial Neural Network, adaptive neuro-fuzzy inference system, and gene expression programming) for linking hydro-social science and water science; (2) estimation of several social variables (rural society, society, technology and communication, and knowledge) in function of the water resources of the continents, and estimation of water resources in terms of social variables; (3) mathematical functions for social parameters are shown to be scalable in space and time.

## Methodology

### Selected indicators

The renewable water per capita (RWPC) is chosen as the overall indicator of water resource status. The indicators corresponding to rural society, urban society, technology and communication, and knowledge are the ratio of rural to urban population (PRUP), population density (PD), number of internet users (IU), and education index (EI), respectively; each of them is defined below.

- Renewable water per capita (*RWPC*): Renewable water is the amount of water that a basin can replenish during the annual water cycle. Per capita renewable water is the available volume of renewable water per person every year measured in millions of cubic meters per person.
- The ratio of rural to urban population (*PRUP*): This index compares the number of people living in rural areas to the number of people living in urban areas (a rural population division into the urban population). The ratio of rural to urban population (*PRUP*) is herein proposed as a potential indicator of the water resources' status.
- Population density (*PD*): Population density measures the number of inhabitants per unit area. The unit of this parameter is persons per square kilometer. Several authors have applied population density as an indicator of the status of water resources^28–40^.
- Internet users (*IU*): The number of people who have access to the Internet and use it for their daily work. This indicator is effective in mass information related to water use. The unit of this parameter is the percentage of the internet-using population with respect to the total population. The term "Water Internet" is reminiscent of water use and internet connectivity. The Water Internet is a source of water supply information for involved organizations and citizens in general^22,34,39^.
- Education index (*EI*): The educational level is a leading determinant of a person’s knowledge about the use of water resources. The Education index (*EI*) is calculated as the average years of schooling received by a population of individuals^34^.

**Figure 1:**
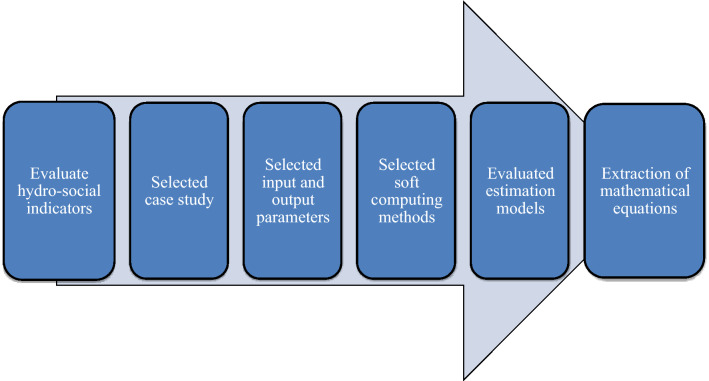
The phases of this work’s methodology.

**Figure 2:**
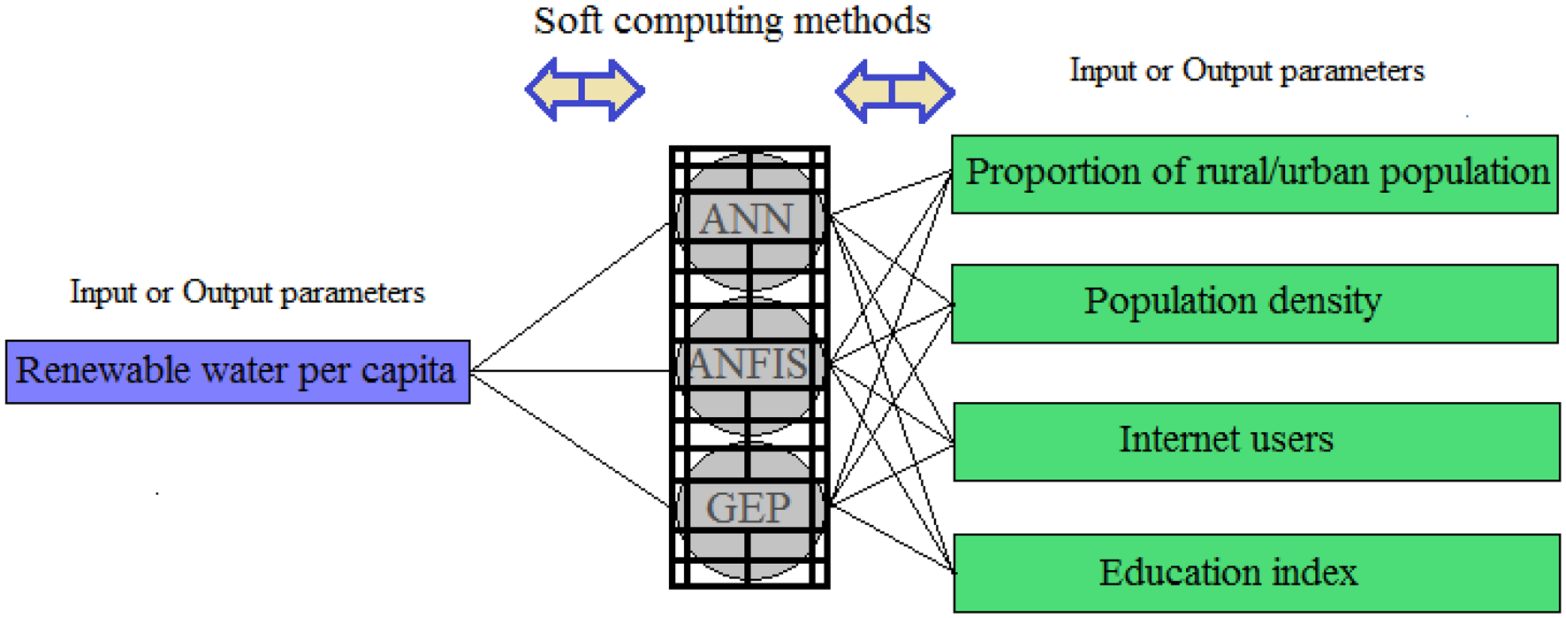
The components of this paper’s methodology.

Figure 1 displays the phases of this paper's methodology. This work proposes the social indicators *PRUP*, *PD*, *IU*, and *EI* to quantify the *RWPC* in Africa, America, Asia, Europe, and Oceania with soft computing methods (ANN, ANFIS-SC, and GEP). This paper analysis evaluated two types of functional patterns of associations: (1) per capita data on water resources were applied as input and values of social parameters were quantified as output; (2) social parameters were applied as input and per capita water resources parameter were quantified as output. The components of this paper’s methodology are shown in Fig. 2.

The per capita renewable water data and social indicators were normalized for each country with Eq. (1). Normalized values range between 0 and 1.

1 $$ X_{N} = \frac{{(X_{i} - X_{\min } )}}{{(X_{\max } - X_{\min } )}} $$

where *X*_*N*_, *Xi*, *Xmin*, and *Xmax* denote the normalized value, the real value, minimal value, and the maximal value, respectively. Normalization operations are performed before the modeling process so that the algorithm fairly examines the various dimensions of the databased on the same standardized range. This work implements a normalization process with Eq. (1) for training and testing^41,42^.

**Figure 3:**
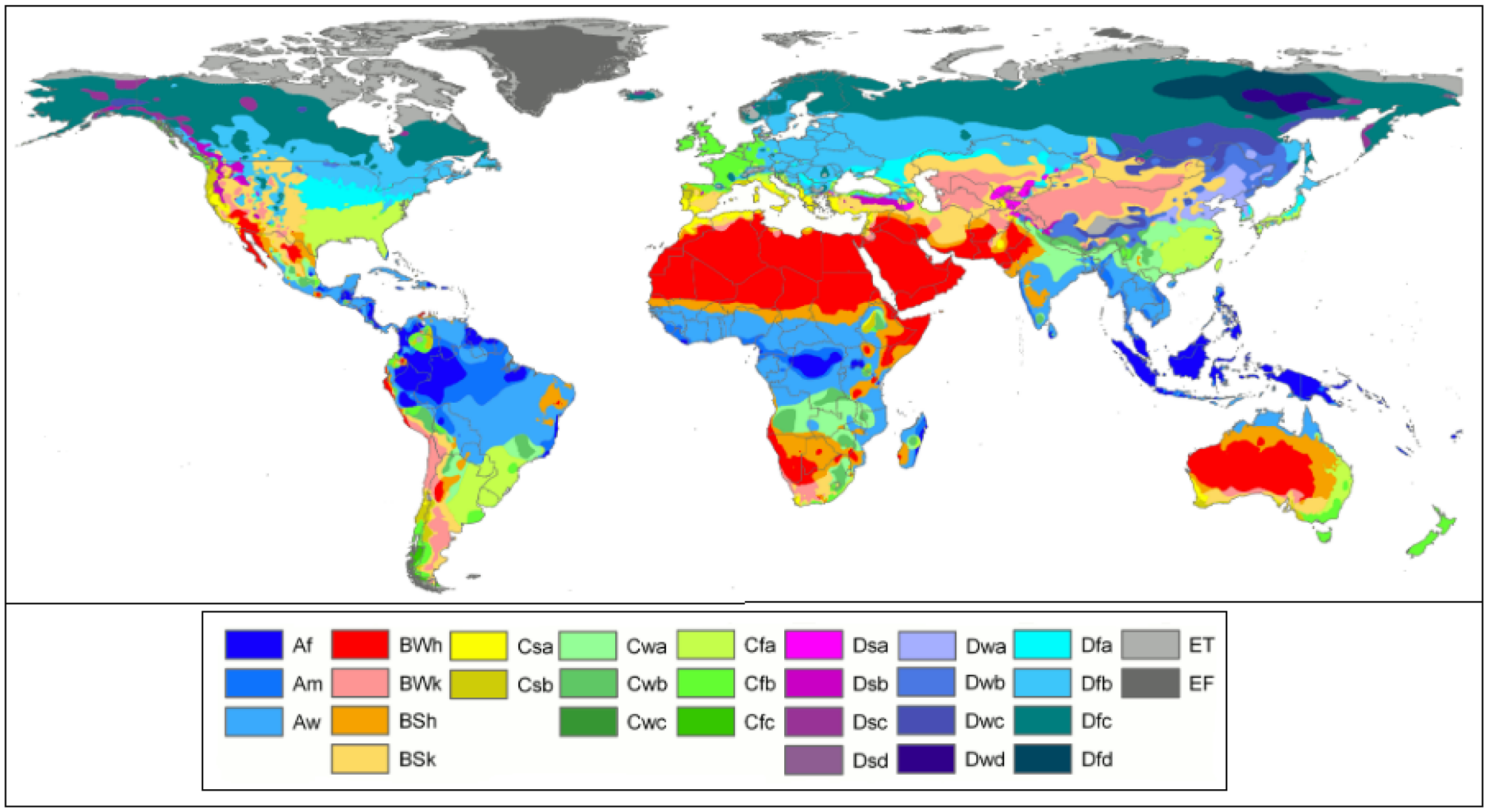
The Köppen climate classification of the world. (Zone A: tropical or equatorial zone; Zone B: arid or dry zone; Zone C: warm/mild temperate zone; Zone D: continental zone; Zone E: polar zone)^59^.

This study selected countries for analysis that exhibit a decreasing trend of per capita renewable water in the 13-year study period (2005–2017). The studied countries' names classified by continent are listed in Table 1. Africa, America, Asia, Europe, and Oceania include 48, 35, 43, 20, and 5 countries across numerous climate conditions, respectively. This study randomly chose 70% of each continent's countries for model training and 30% for model testing. This work relied on data extracted from the Knoema database (https://knoema.com). The statistical characteristics such as average, standard deviation, and coefficient of variation of the hydro-social indicators are listed in Table 2. The coefficients of variation of per capita renewable water equaled 0.663, 0.654, 0.683, 0.329, and 0.640 for Africa, America, Asia, Europe, and Oceania, respectively. Europe has the smallest coefficient of variation of PRUP, PD, IU, and EI indicators. The effect of the Köppen climatic classification was herein considered to examine the patterns of association between hydro-social indicators. Figure 3 displays the Köppen climate classification of the world. The Köppen climate classification scheme^43^ divides the climates into five main groups (A, B, C, D, and E); each group can be further classified by precipitation and temperature conditions.

**Table 1:** Countries included in this study in the training and testing periods.

*s*	Continent		Countries
1	Africa	Train	Algeria, Angola, Benin, Botswana, Burkina Faso, Burundi, Cabo Verde, Cameroon, Central African Republic, Chad, Comoros, Congo, Côte d'Ivoire, Djibouti, Equatorial Guinea, Eswatini, Ethiopia, Gabon, Gambia, Ghana, Guinea, Guinea Bissau, Kenya, Lesotho, Liberia, Libya, Madagascar, Malawi, Mali, Mauritania, Mauritius, Mozambique, Namibia, Niger
		Test	Nigeria, Rwanda, Sao Tome and Principe, Senegal, Sierra Leone, South Africa, Sudan, Togo, Tunisia, Uganda, United Republic of Tanzania, Zambia, Zimbabwe, Morocco
2	America	Train	United States of America, Mexico, Canada, Cuba, Guatemala, Haiti, Honduras, Dominican Republic, El Salvador, Costa Rica, Nicaragua, Panama, Jamaica, Trinidad and Tobago, Bahamas, Belize, Barbados, Saint Lucia, Saint Vincent and the Grenadines, Grenada, Antigua and Barbuda, Dominica, Argentina, Bolivia, Brazil
		Test	Chile, Colombia, Ecuador, Guyana, Paraguay, Peru, Saint Kitts and Nevis, Suriname, Uruguay, Venezuela
3	Asia	Train	Afghanistan, Azerbaijan, Bahrain, Bangladesh, Brunei Darussalam, Cambodia, China, Cyprus, Egypt, India, Indonesia, Iran, Iraq, Israel, Jordan, Kazakhstan, Kyrgyzstan, Lao People’s Democratic Republic, Lebanon, Malaysia, Maldives, Mongolia, Myanmar, Nepal, Oman, Pakistan, Papua New Guinea, Philippines, Qatar, Russian Federation
		Test	Saudi Arabia, Singapore, Sri Lanka, Tajikistan, Thailand, Timor-Leste, Turkey, Turkmenistan, United Arab Emirates, Uzbekistan, Viet Nam, Yemen, Palestine
4	Europe	Train	Austria, Belgium, Czech Republic, Denmark, Finland, France, Germany, Iceland, Ireland, Italy, Luxembourg, Malta, Netherlands, Norway
		Test	Slovakia, Slovenia, Spain, Sweden, Switzerland, United Kingdom
5	Oceania	Train	Australia, Fiji, New Zealand
		Test	Solomon Islands, Vanuatu

**Table 2:** The statistical characteristics of hydro-social indicators.

Continents	Characteristics	Indicators				
		*PRUP* (P_1_)	*PD* (P_2_)	*IU* (P_3_)	*EI* (P_4_)	*RWPC*
Africa	Mean	0.512	0.478	0.393	0.582	0.473
	S	0.328	0.317	0.351	0.343	0.314
	CV	0.640	0.663	0.894	0.589	0.663
America	Mean	0.525	0.498	0.499	0.550	0.484
	S	0.335	0.318	0.335	0.342	0.317
	CV	0.638	0.638	0.671	0.621	0.654
Asia	Mean	0.504	0.486	0.462	0.559	0.474
	S	0.338	0.328	0.352	0.351	0.324
	CV	0.670	0.675	0.762	0.628	0.683
Europe	Mean	0.496	0.525	0.621	0.564	0.475
	S	0.329	0.330	0.310	0.345	0.329
	CV	0.329	0.330	0.310	0.345	0.329
Oceania	Mean	0.467	0.472	0.482	0.579	0.492
	S	0.312	0.323	0.337	0.337	0.315
	CV	0.668	0.684	0.700	0.582	0.640

### Soft-computing models

Soft computing methods are effective in detecting new and valuable information from large datasets with the purpose of discovery, classification, and forecasting^44^. The well-known ANNs, ANFIS, and GEP are described in the next section.

### ANNs

Artificial neural networks (ANNs) are computing systems inspired by biological neural networks. The initial aim of neural networks was to solve complex problems mimicking the human mind. Over time ANNs' focus shifted to emulating specific mental abilities. An ANN is based on a set of connected units or nodes, called artificial neurons (similar to biological neurons in the animal brain). Any synapse between neurons can transmit a signal from one neuron to another. The receiving neuron can process the signals. In conventional ANN the synapse signal is a real number, and a nonlinear function of its inputs calculates each neuron's output. Neurons and synapses apply weights that are adjusted as learning progresses. This weight increases or decreases the signal strength that it sends to the synapse. Neurons are commonly organized in layers. The signals travel from the first layer (input) to the last layer (output), and they may travel multiple times^45–47^. Three fundamental characteristics of ANNs for determining an optimal solution are: (1) the applied algorithm, (2) the activation functions, and (3) the neurons, as follows:

1. The applied algorithm and layer characteristics: The Levenberg–Marquardt (LM) algorithm with three-layer has been selected for use in this study due to its faster convergence in training networks. The error propagation algorithm changes the network weights and bias values so that the activation function decreases more rapidly.
2. Activation Functions: Selecting the activation function has a significant effect on the accuracy of the network output. There are three main activation functions for neural network modeling: the Logsig, Tansig, and Purelin functions. Several activation functions are used to develop the network to achieve the best combination of activation functions in a network with one to three hidden layers. The log-sig, tan-sig, and pure-line functions were applied in the hidden and output layers. Using one or a combination of these activation functions (between layers) may lead to an optimal model with the highest correlation value and the smallest error.
3. The neuron number determination: Determining the number of hidden layers to create a network with the least error in predicting the desired outputs is essential. Trial-and-error is the best way to determine the optimal number of neurons in the hidden layer of ANN models^48–50^. The number of neurons in the lattice layer has a significant effect on the neural network's function. Using a small number of neurons prevents the neural network from learning most of the patterns accurately. On the other hand, the presence of a large number of neurons leads to the preservation of patterns and thus prevents the neural network from learning to recognize their basic features.

The authors wrote the ANN program code in MATLAB.

### ANFIS

A neural-fuzzy inference system is an artificial neural network based on the Takagi–Sugeno fuzzy system^51^, which is in accordance with the set of fuzzy rules if–then that learns to identify nonlinear fitting functions. ANFIS is a universal estimator^52^, which is herein applied. If–Then fuzzy rules are required to specify functions between the fuzzy variables of a fuzzy system. Equations (2) and (3) give a typical rule set with two fuzzy If–Then rules in a first order Sugeno system:

2 $$ {\text{Rule}}\,1:\,{\text{If}}\,{\text{x}}\, = {\text{A}}1\,{\text{and}}\,{\text{y}} = {\text{B}}1,\,{\text{then}}\,{\text{f}}1 = {\text{p}}1{\text{x}} + {\text{q}}1{\text{y}} + {\text{r}}1 $$

3 $$ {\text{Rule}}\,2:\quad {\text{If}}\,x = {\text{A}}2\,{\text{and}}\,{\text{y}} = {\text{B}}2,\,{\text{then}}\,{\text{f}}1 = {\text{p}}2{\text{x}} + {\text{q}}2{\text{y}} + {\text{r}}2 $$

where A1 (LOW), A2 (LOW), and B1 (HIGH), B2 (MEDIUM) denote the membership functions (MFs) for inputs x and y, respectively, the importance of the clusters’ number of ANFIS-SC is determining the efficient radius amount based on the trial- and-error method. The radius varies from 0.20 to 0.60^42^.

The ANFIS method with Subtractive Clustering (ANFIS-SC) is herein applied. Subtractive Clustering (SC) is an extension of the mountain clustering method proposed by Yager and Filev^53^, in which the data are clustered by evaluating the potential of data in the specification space^54^. Linear least squares (LLS) are applied to determine the MF's output, following previous works^42,50,55^ These authors wrote the code for ANFIS-SC in MATLAB.

### GEP

Gene expression programming is a method of mathematical modeling based on evolutionary computation and inspired by natural evolution. This method was introduced by Ferreira^56^ in 1999 and advanced in 2001. The GEP algorithm integrates the dominant view of the two predecessor inheritance algorithms to resolve their weaknesses. GEP features a chromosome genotype similar to a genetic algorithm (Genetic Algorithm), and the phenotype of a chromosome has a tree structure with length and size variable similar to the genetic programming algorithm^56^. Design and implementation steps of GEP are: (1) Defining the fitness function; (2) Defining the terminals and functions; (3) Determining the structure of chromosomes (number of generations, number, and length of genes); (4) Determining the Linking Function of Genes; (5) Specifying the operators, and executing the algorithm^56^. The fundamental characteristic of GEP for determining the optimal model or fitting functions are the mutation rate (MR), the inversion rate (IR), the IS transposition rate (ISTR), and the RIS transposition rate (RISTR), one-point recombination rate (OPRR), two-point recombination rate (TPRR), gene recombination rate (GRR), and gene transposition rate (GTR) whose values are listed in Table 3. The penalizing tool with parsimony pressure was applied in this study, which. implements several mathematical functions to predict the hydro-social indicators $( + , - ,*,/,\ln x,e^{x} ,x^{2} ,x^{3} ,\sqrt x ,\sqrt[3]{x},\sin x,\cos x,\arctan (x))$.

**Table 3:** The GEP model parameters.

**General characteristics**	
Linking function	Summation
Fitness function error type	RRSE
**Genetic operator values**	
MR	0.044
IR	0.1
ISTR	0.1
RISTR	0.1
OPRR	0.3
TPRR	0.3
GRR	0.1
GTR	0.1

This study first employs the terminal set for the renewable water per capita indicator with output sets containing *PRUP*, *PD*, *IU,* and *EI,* and then employs the terminal sets for the cited social indicator with output renewable water per capita indicator. This study employs the soft-computing software GeneXpro Tools 4.0.

### Evaluating model performance

The goodness-of-fit correlation coefficient (*R*), root mean squared error (*RMSE*), and mean absolute error (*MAE*) were applied to evaluate the model's performance. The *R*, *RMSE*, and *MAE* are respectively defined as follows:

4 $$ R = \left[ {\frac{{\sum\limits_{i = 1}^{N} {(hydrosocio_{io} - \overline{hydrosocio}_{o} )(hydrosocio_{ie} - \overline{hydrosocio}_{e} )} }}{{\sqrt {\sum\limits_{i = 1}^{N} {(hydrosocio_{io} - \overline{hydrosocio}_{o} )^{2} \sum\limits_{i = 1}^{N} {(hydrosocio_{ie} - \overline{hydrosocio}_{e} )^{2} } } } }}} \right] $$

5 $$ RMSE = \sqrt {\frac{1}{N}\sum\limits_{i = 1}^{N} {(hydrosocio_{io} - hydrosocio_{ie} )^{2} } } $$

6 $$ MAE = \frac{1}{N}\sum\limits_{i = 1}^{N} {\left| {hydrosocio_{io} - hydrosocio_{ie} } \right|} $$

where $\overline{hydrosocio}_{o}$ and $\overline{hydrosocio}_{e}$ denote the average observed and estimated hydro-social indicators’ values respectively, *hydrosocio*_*io*_ and *hydrosocio*_*ie*_ denote the observed and estimated hydro-social indicators’ values respectively, and *N* denotes the number of data.

The correlation coefficient (*R*) measures the degree of statistical association (positive or negative) between variables. The *RMSE* measures the goodness of fit, giving higher weight to high values of observations. by comparing the estimated values and the observed values. The *MAE* measures the distribution of goodness of fit at moderate values^57^. The models’ performances are optimal if the *R* and *RMSE* are closer to 1 and 0, respectively. This study also employs various graphical methods to display models’ results.

## Results and discussion

### Evaluating indicators

Among the selected parameters the ratio of rural to the urban population directly relates to the per capita renewable water, whereas the population density, internet users, and education index exhibit an inverse relation with the per capita renewable water worldwide. It means the per capita renewable water decreases with decreasing rural to urban population and increasing population density, internet users, and education index. The urban population has increased in developing regions, which feature increasing population density. People's health is threatened by poor urban sanitary infrastructure leading to disease and social decay. Increasing population density and a reduction in per capita renewable water inflict social harm and disrupt society's economic growth^58^. Population density also is positively related to the relative number of elderly and social vulnerability because potential casualties increase with population size^40^. On the other hand, with the increase of Internet users and education index, the per capita renewable water has increased. As long as the knowledge and awareness of communities improved, the consumption algorithm decreased, leading to a reduction of renewable water per capita. Therefore, the level of literacy and knowledge for a community can be the basis for making the right decisions in agriculture, health, natural resource management, and other activities related to water resources for decision-makers. The latter situation calls for better communication among water users through social media and improved education to learn and develop optimal water management.

### Evaluating models and developing hydro-social equations

Three soft-computing approaches, namely ANN-LM, ANFIS-SC, and GEP, were applied to develop predictive equations with social indicators worldwide. The ANN-Levenberg–Marquardt (LM) backpropagation algorithm with one hidden layer was applied, and the hidden nodes’ number was determined by trial and error. A hybrid algorithm was combined with the ANFIS-SC models. There is no rule for determining the radii values of the ANFIS-SC models. The final radii values were determined by trial-and-error.

**Figure 4:**
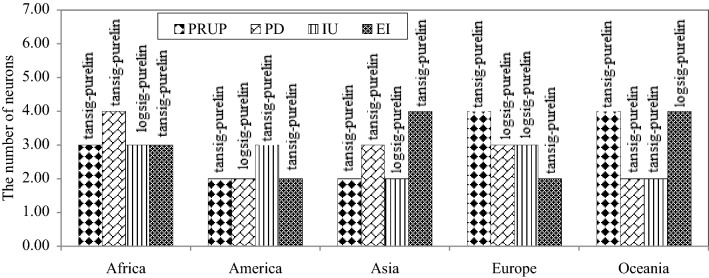
The characteristics of optimal ANN models; showing the number of neurons and activation functions of hidden and output layers.

**Figure 5:**
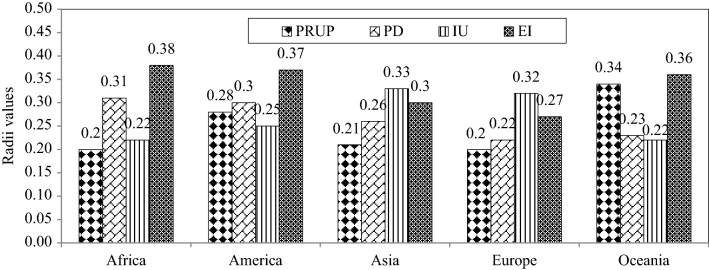
The characteristic of optimal ANFIS-SC model showing the radii values.

The numbers of neurons in the ANN-LM models and the radii values of the ANFIS-SC models are listed in Table 4. The activation functions of the output nodes were linear for all the continents. The activation functions of the hidden nodes of the ANN-LM models for the P1 through P4 indicators were respectively the tangent sigmoid, tangent sigmoid, tangent sigmoid, and logarithm sigmoid for Africa; the activation functions of the proportion of rural to urban population was the tangent sigmoid for all the continents. Table 5 lists the results of the soft computing optimal models' estimates of the proportion of rural to urban population (*PRUP*), population density (*PD*), internet users (*IU*), and education index (*EI*), denoted respectively by P1 through P4, during the test period in the world's continents. Figures 4 and 5 display the characteristics of ANN (the number of neurons and activation functions of hidden and output layers) and ANFIS-SC (radii values) models, respectively. The values of *R* and *RMSE* for Africa corresponding to the ANN-LM models were respectively (0.921, 0.981, 0.858, 0.862) and (0.193, 0.058, 0.190, 0.172) associated with the *PRUP*, *PD*, *IU*, and *EI* parameters, respectively. The values of *R* and *RMSE* for Africa corresponding to the ANFIS-SC models equaled respectively (0.933, 0.991, 0.868, 0.891) and (0.130, 0.044, 0.186, 0.156) for the P1 through P4 parameters, respectively. Concerning the GEP models, the root relative squared error (*RRSE*) was selected as the pressure tree's fitness function. The values of *RMSE* for GEP models equaled (0.084, 0.029, 0.178, 0.135), (0.197, 0.056, 0.152, 0.163), (0.151, 0.036, 0.123, 0.210), (0.182, 0.039, 0.148, 0.204) and (0.141, 0.030, 0.226, 0.082) for Africa, America, Asia, Europe, and Oceania, respectively. Table 5 results for the *R*, *RMSE*, and *MAE* values establish the GEP model estimates of *PRUP*, *PD*, *IU*, and *EI* indicators had the highest *R* values and the lowest *RMSE* values. The average *R* values of the best models (GEP) for all selected social parameters equaled 0.942, 0.909, 0.910, 0.889, and 0.947 for Africa, America, Asia, Europe, and Oceania, respectively. These results indicate the climatic characteristics of the continents influence the performance of the models. The models' performances for Africa and Oceania associated with the type *B* dominant Koppen climate classification was the best. The models' performances for Asia and America that have similar climatic classification were nearly equal. The average model performance for Europe in the type *D* climate classification was the poorest among the continents.

**Table 4:** The characteristics of ANN (the number of neurons) and ANFIS (radii values) models corresponding to social indicators and continents.

Indicators (P_i_)		Africa	America	Asia	Europe	Oceania
P_1_	ANN-LM	3	2	2	4	4
	ANFIS-SC	0.20	0.28	0.21	0.20	0.34
P_2_	ANN-LM	4	2	3	3	2
	ANFIS-SC	0.31	0.30	0.26	0.22	0.23
P_3_	ANN-LM	3	3	2	3	2
	ANFIS-SC	0.22	0.25	0.33	0.32	0.22
P_4_	ANN-LM	3	2	4	2	4
	ANFIS-SC	0.38	0.37	0.30	0.27	0.36

**Table 5:** The results of soft computing optimal models corresponding to the testing period in the world’s continents.

Indicators (P_i_)	Model	Africa			America		
		*R*	*RMSE*	*MAE*	*R*	*RMSE*	*MAE*
*PRUP* (P_1_)	ANN-LM1	0.921	0.193	0.101	0.673	0.252	0.195
	ANFIS-SC1	0.933	0.130	0.082	0.778	0.220	0.178
	GEP1	0.972	0.084	0.061	0.837	0.197	0.166
*PD* (P_2_)	ANN-LM2	0.981	0.058	0.038	0.960	0.090	0.054
	ANFIS-SC2	0.991	0.044	0.029	0.979	0.066	0.037
	GEP2	0.998	0.029	0.021	0.985	0.056	0.030
*IU* (P_3_)	ANN-LM3	0.858	0.190	0.131	0.849	0.190	0.130
	ANFIS-SC3	0.868	0.186	0.125	0.894	0.172	0.108
	GEP3	0.878	0.178	0.117	0.917	0.152	0.094
*EI* (P_4_)	ANN-LM4	0.862	0.172	0.132	0.832	0.198	0.156
	ANFIS-SC4	0.891	0.156	0.118	0.868	0.180	0.141
	GEP4	0.921	0.135	0.094	0.896	0.163	0.130

Indicators (P_i_)	Model	Asia			Europe		
		*R*	*RMSE*	*MAE*	*R*	*RMSE*	*MAE*
*PRUP* (P_1_)	ANN-LM1	0.824	0.191	0.142	0.725	0.237	0.183
	ANFIS-SC1	0.834	0.188	0.14	0.800	0.206	0.157
	GEP1	0.899	0.151	0.114	0.856	0.182	0.149
*PD* (P_2_)	ANN-LM2	0.991	0.047	0.034	0.980	0.070	0.043
	ANFIS-SC2	0.993	0.040	0.03	0.991	0.047	0.030
	GEP2	0.995	0.036	0.025	0.994	0.039	0.023
*IU* (P_3_)	ANN-LM3	0.925	0.131	0.103	0.809	0.183	0.136
	ANFIS-SC3	0.933	0.127	0.102	0.834	0.171	0.122
	GEP3	0.936	0.123	0.096	0.878	0.148	0.105
*EI* (P_4_)	ANN-LM4	0.797	0.217	0.163	0.762	0.234	0.183
	ANFIS-SC4	0.803	0.214	0.159	0.787	0.224	0.177
	GEP4	0.810	0.210	0.155	0.826	0.204	0.160

Indicators (P_i_)	Model	Oceania					
		*R*	*RMSE*	*MAE*			
*PRUP* (P_1_)	ANN-LM1	0.834	0.181	0.136			
	ANFIS-SC1	0.874	0.165	0.129			
	GEP1	0.905	0.141	0.106			
*PD* (P_2_)	ANN-LM2	0.982	0.081	0.063			
	ANFIS-SC2	0.989	0.063	0.041			
	GEP2	0.997	0.030	0.024			
*IU* (P_3_)	ANN-LM3	0.880	0.246	0.206			
	ANFIS-SC3	0.887	0.230	0.190			
	GEP3	0.905	0.226	0.182			
*EI* (P_4_)	ANN-LM4	0.95	0.118	0.082			
	ANFIS-SC4	0.965	0.103	0.078			
	GEP4	0.981	0.082	0.059

**Figure 6:**
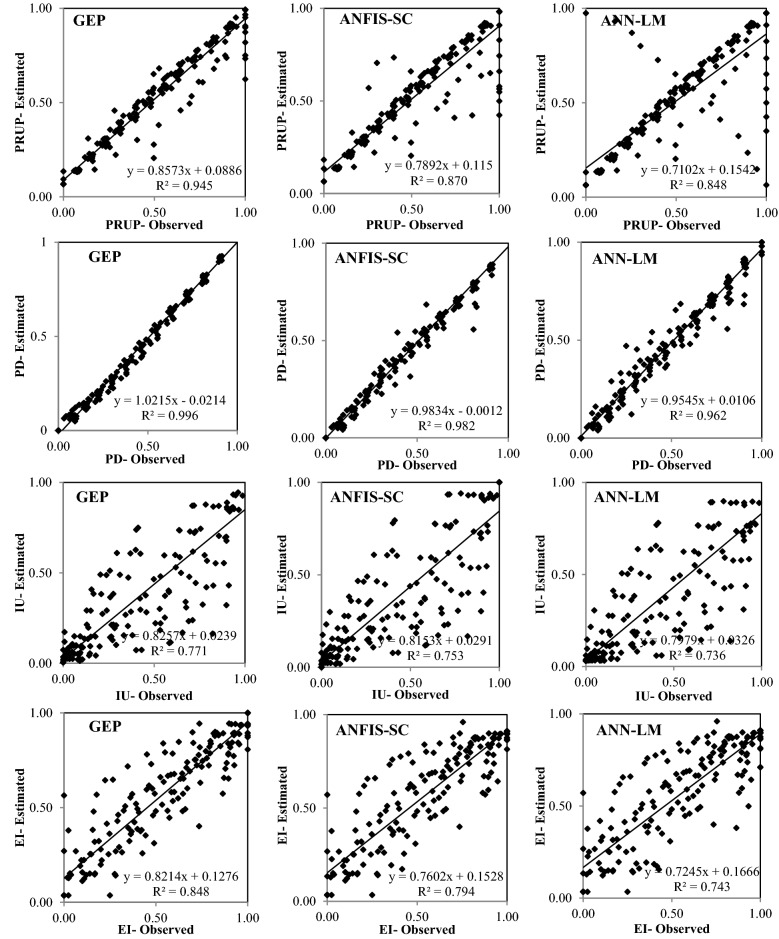
Observed and estimated social parameters during the testing period in Africa.

**Figure 7:**
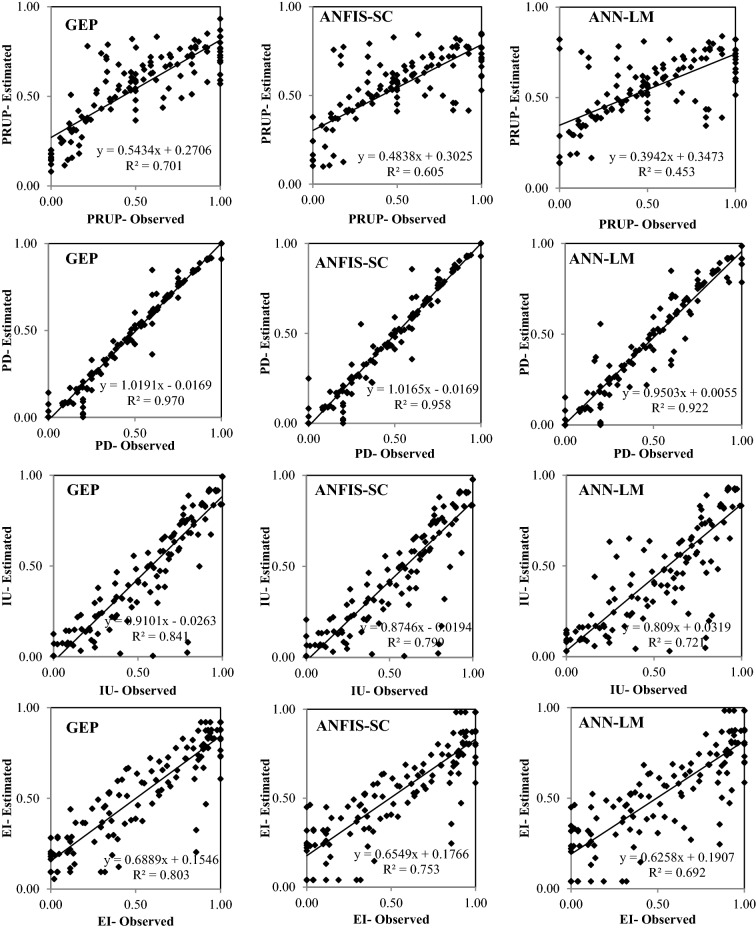
Observed and estimated social parameters during the testing period in America.

**Figure 8:**
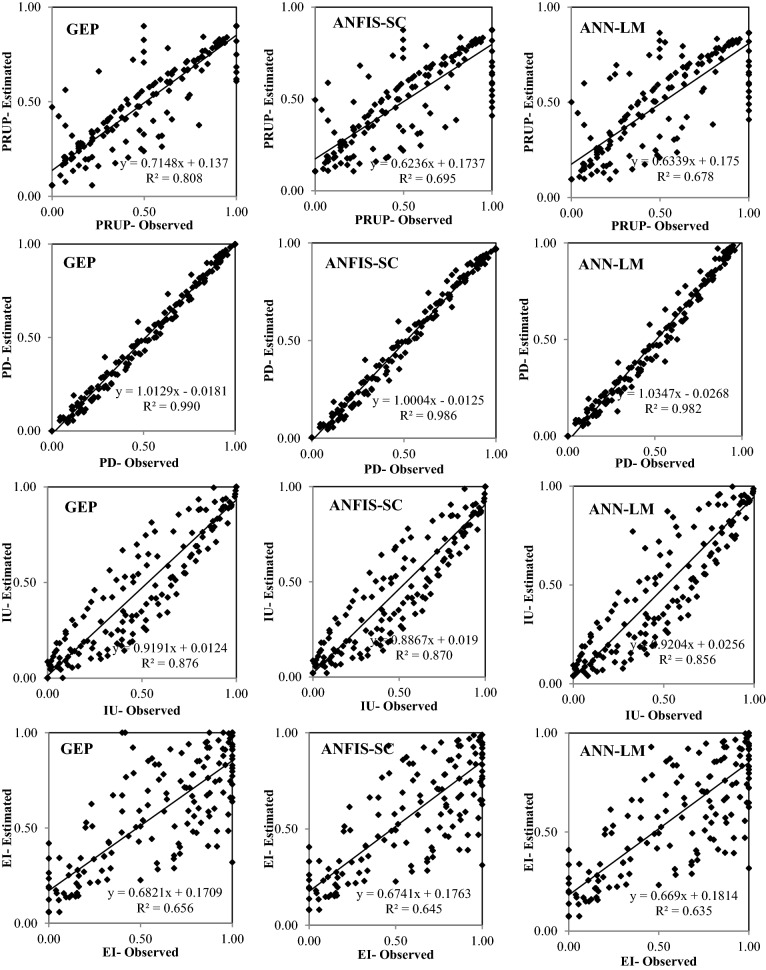
Observed and estimated social parameters during the testing period in Asia.

**Figure 9:**
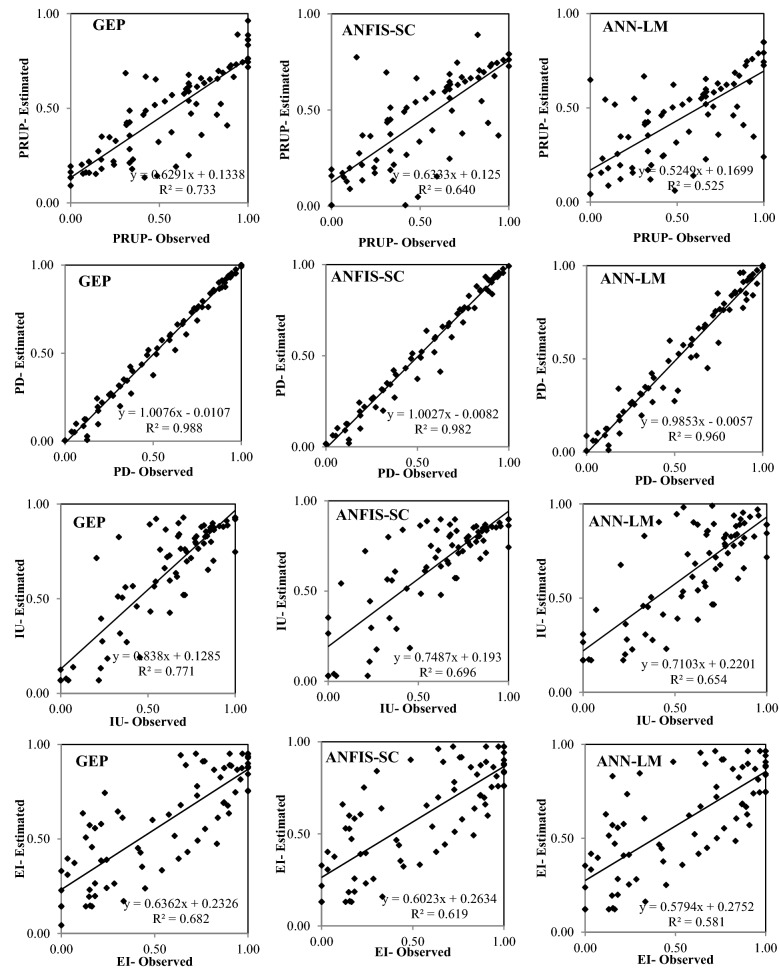
Observed and estimated social parameters during the testing period in Europe.

**Figure 10:**
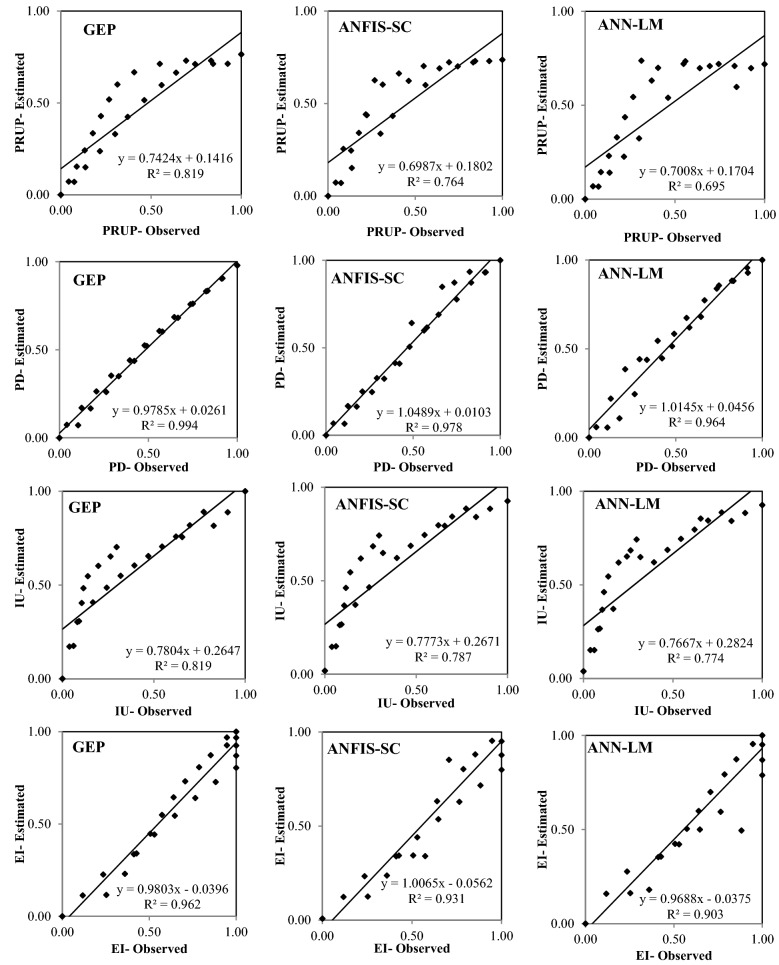
Observed and estimated social indicators during the testing period in Oceania.

**Figure 11:**
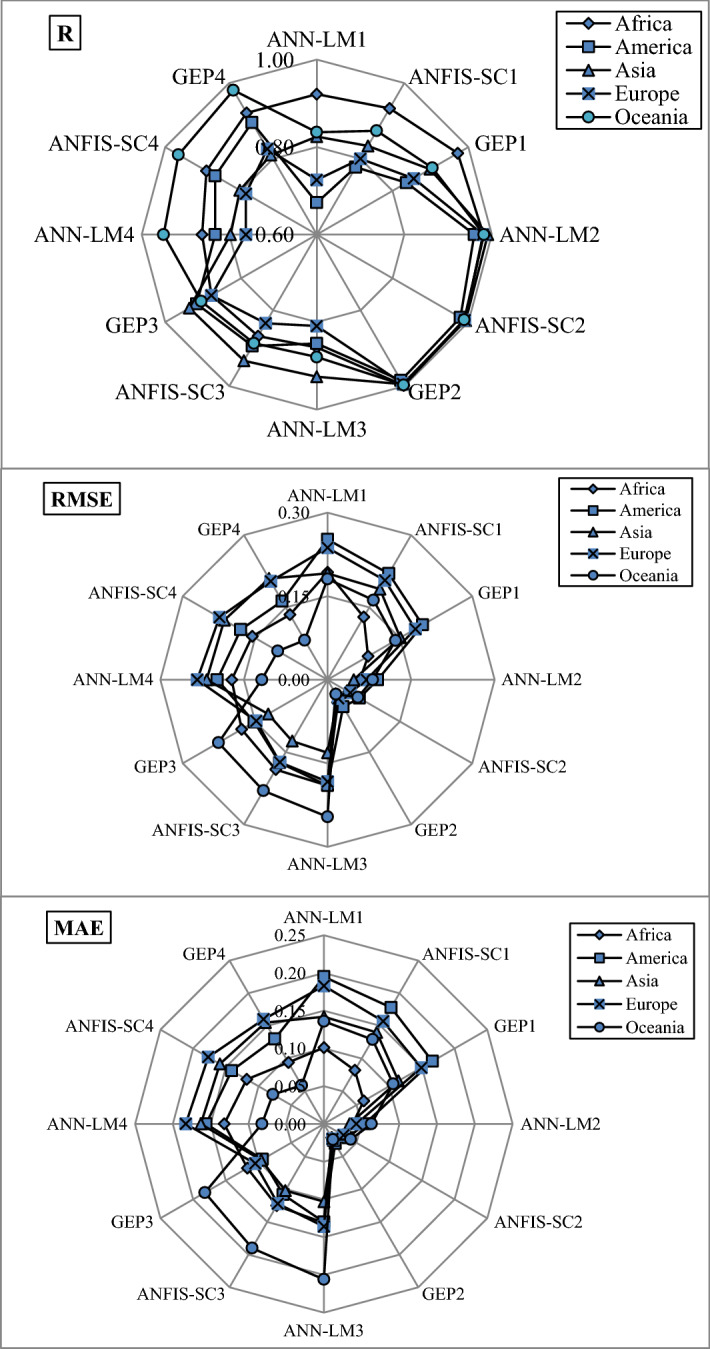
Comparison of *R*, *RMSE* and *MAE* values corresponding to the soft computing methods.

Figures 6, 7, 8, 9 and 10 show the observed and estimated social parameters obtained with the soft-computing models during the test period in Africa, America, Asia, Europe, and Oceania, respectively. Figure 11 compares the R, RMSE, and MAE values from the soft-computing models. The R values for soft-computing models are close to 1, with the quality relations being: RGEP > RANFIS-SC > RANN-LM for all social indicators. Figure 11 establishes that the ANFIS-SC model exceeded the ANN-LM models’ performance. Also, the GEP models had better performance than the ANFIS-SC and ANN-LM for estimating the proportion of rural to urban population (PRUP), population density (PD), internet users (IU), and education index (EI) parameters in Africa, America, Asia, Europe, and Oceania.

The main advantage of the GEP over other soft computing methods (e.g., ANFIS and ANN) is in producing predictive equations. The equations obtained with the optimal models for the social indicators (i.e., the proportion of rural to urban population (PRUP), population density (PD), internet users (IU), and education index (EI) in Africa, America, Asia, Europe, and Oceania) are listed in Table 6. The equations that the GEP model discovers as a structure do not necessarily correspond to reality. The equations listed in Table 6 merely show the optimal equations extracted from the model after the evolution, for all indicators and in all basins (considering renewable water per capita as a decision variable).

**Table 6:** Mathematical equations governing hydro-social indicators.

Continent	Equations
Africa	$PRUP_{ts} = [H_{ts}^{2} \cos (H_{ts} \cos (H_{ts} ))]^{1/2} + 0.07$
	$$PD_{ts} = [\sin (0.45 - H_{ts} )\cos (6.1H_{ts} )]^{3} + H_{ts} (0.84 + H_{ts} (H_{ts} - 1)) + 0.94$$
	$$IU_{ts} = \exp [A\tan (( - 7.03)(3H_{ts} + 2.49))] + A\tan ( - 2H_{ts} ) + \cos (A\tan (A\tan (\cos (H{}_{ts})))$$
	$$EI_{ts} = (\sin (A\tan (H_{ts} )))[A\tan (\sin (H_{ts} ))]^{3} + 0.03(A\tan (18.15H_{ts} )) + [\cos (A\tan (H_{ts} ))^{1/3} ]\cos (H_{ts} (H_{ts} + 1))$$
America	$PRUP_{ts} = - 0.46[\sin (\sin (H_{ts}^{2} ))] + H_{ts} + 0.18$
	$$PD_{ts} = \cos (A\tan (H_{ts} ) + H_{ts}^{1/3} ) + H_{ts} [H_{ts}^{1/3} + A\tan (H_{ts} )]$$
	$$IU_{ts} = [0.33A\tan (H_{ts}^{1/3} )]\cos (7.87 + H_{ts} ) + \sin (H_{ts} - 9.33) + [A\tan (3.62 + 2H_{ts} )]^{1/3}$$
	$$EI_{ts} = 0.61(1 - H_{ts} ) + 0.47(\cos (H_{ts} ) - 0.47) + 0.67$$
Asia	$PRUP_{ts} = [\sin (H_{ts} ) - H_{ts} ] + \sin [[\ln (A\tan (\cos (H_{ts}^{3} - H_{ts} )))]^{2} ] + Ln[\exp (H_{ts}^{1/3} ) + (H_{ts} (H_{ts} - 1))]^{2}$
	$$PD_{ts} = \cos [H_{ts}^{3} - (H\sin (H_{ts} ))^{2} ] + A\tan (A\tan (( - 1.14)\sin (H_{ts} ))] - 0.13H_{ts} - \sin (H_{ts} ) + A\tan (A\tan (H_{ts} ))$$
	$$IU_{ts} = A\tan (\sin (H_{ts}^{3} ) - H_{ts} )^{3} + \cos (H_{ts}^{1/2} ) - H_{ts} + 0.61H_{ts} \sin (H_{ts} )$$
	$$EI_{ts} = H_{ts}^{4/3} + \cos (H_{ts} ) - 1.48H_{ts}$$
Europe	$PRUP_{ts} = H_{ts} + [A\tan (\cos (H_{ts} ))]^{2} + 1.69$
	$$PD_{ts} = \sin [(H_{ts} (1 - H_{ts} )\sin (H_{ts} )]^{3} + [H_{ts}^{4} - \sin (H_{ts}^{2} )]^{3} - H_{ts} + 1$$
	$$IU_{ts} = [\exp ((H_{ts} - 2.97) - 8.25H_{ts} )]\sin (( - 8.25H_{ts} ) - 0.34(\frac{{\exp (H_{ts} )}}{{\cos (H_{ts} )}})\cos (A\tan (H_{ts} )) + 1.26$$
	$$EI_{ts} = (H_{ts} + 1.36)^{1/6} - A\tan (H_{ts} ) + [\cos (\cos (4.45 + H_{ts}^{2} ))]^{1/2} + (1 - H_{ts} )(6.72 + H_{ts} )\exp (H_{ts} - 5.65) - 1.11$$
Oceania	$PRUP_{ts} = (\sin (H_{ts} ))^{2} + H_{ts}^{2} \sin (H_{ts} ) - A\tan (H_{ts} )$
	$$PD_{ts} = A\tan (8.77 - (2H_{ts} )^{1/3} + H_{ts}^{3} ) - H_{ts} - 0.48$$
	$$IU_{ts} = Ln(\cos (H_{ts}^{1/2} )) + \cos ((\sin (\sin (H_{ts} )))^{1/3} ) + H_{ts}^{2} (1 - H_{ts} )$$
	$$EI_{ts} = ( - 0.55)A\tan (0.75H_{ts} ) - 0.25A\tan (H_{ts} \sin (2H_{ts} )) + \cos (H_{ts} )$$

*H* denotes Hydro: renewable water per capita.

The performance of the GEP models in estimating the social indicators in three ranges of values, namely, 20% of the maximum estimated values (20%max), 60% of median estimated values (60%mid or 20%min to 20%max), and 20% of minimum estimated values (20%min), during the test period for the proportion of rural to urban population (PRUP), population density (PD), internet users (IU) and the education index (EI) parameters of Africa, America, Asia, Europe, and Oceania are listed in Table 7. Table 7’s results indicate there is not a regular rule to determine the best-cited ranges performances. The education index and the population density have the lowest and highest R values among the other parameters in the three different ranges (20%max, 60%mid, and 20%min) in Africa, America, Asia, Europe, and Oceania. Therefore, the results indicate a strong pattern of association between the population density parameter and water resources status in all continents of the world.

**Table 7:** The performance of GEP models with respect to selected ranges.

Indicators (P_i_)	Range	Africa			America		
		*R*	*RMSE*	*MAE*	*R*	*RMSE*	*MAE*
*PRUP* (P_1_)	20%_*min*_	0.910	0.073	0.069	0.649	0.177	0.160
	60%_*mid*_	0.886	0.082	0.064	0.335	0.270	0.296
	20%_*max*_	0.554	0.094	0.050	0.203	0.220	0.194
*PD* (P_2_)	20%_*min*_	0.957	0.018	0.012	0.848	0.038	0.022
	60%_*mid*_	0.991	0.036	0.028	0.961	0.068	0.040
	20%_*max*_	0.993	0.018	0.016	0.959	0.020	0.009
*IU* (P_3_)	20%_*min*_	0.637	0.067	0.044	0.724	0.050	0.035
	60%_*mid*_	0.442	0.229	0.189	0.720	0.192	0.129
	20%_*max*_	0.772	0.201	0.112	0.765	0.073	0.032
*EI* (P_4_)	20%_*min*_	0.547	0.165	0.111	0.506	0.159	0.138
	60%_*mid*_	0.748	0.142	0.105	0.708	0.130	0.111
	20%_*max*_	0.588	0.095	0.067	0.447	0.184	0.129

Indicators (P_i_)	Range	Asia			Europe		
		*R*	*RMSE*	*MAE*	*R*	*RMSE*	*MAE*
*PRUP* (P_1_)	20%_*min*_	0.419	0.158	0.114	0.672	0.118	0.105
	60%_*mid*_	0.633	0.147	0.108	0.518	0.177	0.136
	20%_*max*_	0.458	0.149	0.126	0.722	0.228	0.206
*PD* (P_2_)	20%_*min*_	0.907	0.029	0.019	0.808	0.046	0.028
	60%_*mid*_	0.955	0.044	0.035	0.969	0.046	0.030
	20%_*max*_	0.969	0.017	0.011	0.963	0.016	0.012
*IU* (P_3_)	20%_*min*_	0.699	0.070	0.048	0.523	0.072	0.068
	60%_*mid*_	0.669	0.164	0.148	0.673	0.181	0.136
	20%_*max*_	0.743	0.072	0.052	0.419	0.087	0.062
*EI* (P_4_)	20%_*min*_	0.393	0.142	0.106	0.317	0.255	0.202
	60%_*mid*_	0.325	0.232	0.182	0.546	0.208	0.168
	20%_*max*_	0.324	0.221	0.157	0.570	0.151	0.120

Indicators (P_i_)	Range	Oceania					
		*R*	*RMSE*	*MAE*			
*PRUP* (P_1_)	20%_*min*_	0.945	0.073	0.047			
	60%_*mid*_	0.797	0.151	0.112			
	20%_*max*_	0.797	0.192	0.183			
*PD* (P_2_)	20%_*min*_	0.926	0.027	0.020			
	60%_*mid*_	0.995	0.035	0.031			
	20%_*max*_	1.000	0.014	0.012			
*IU* (P_3_)	20%_*min*_	0.945	0.260	0.219			
	60%_*mid*_	0.872	0.232	0.209			
	20%_*max*_	0.999	0.010	0.007			
*EI* (P_4_)	20%_*min*_	1.000	0.003	0.001			
	60%_*mid*_	0.967	0.082	0.068			
	20%_*max*_	0.489	0.093	0.065			

**Figure 12:**
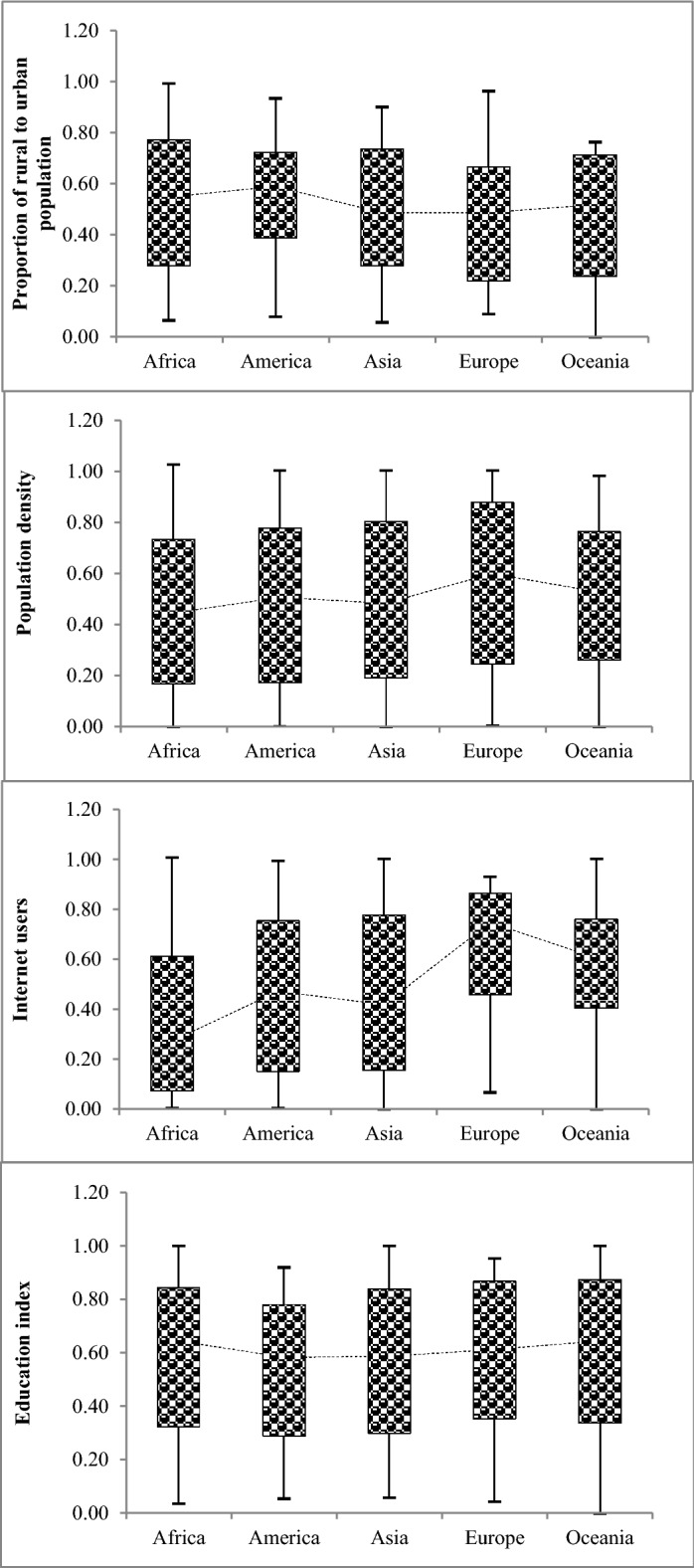
Distribution of estimated data values of social indicators (*P*_*i*_*, i* = 1, 2, …, 4).

Figure 12 depicts the distribution of estimated data values of the social parameters (i = 1, 2, 3, 4) and their comparison through the continents. The box plots are a graphic display integrating multiple numerical relations. One approach to understanding the distribution or dispersion of data is through the box diagram, which is based on the "minimum," "first quartile-Q1(0.25%)", "median (0.50%)", "third quartile-Q3(0.75%)" and "maximum" statistical indicators. Figure 12 shows Oceania and Africa exhibit the smallest and largest values of the rural to urban population, respectively. America has the lowest values of the first to the third quartile. The estimated population density value in Europe has the most values in the third quartile (0.75%). The median values of estimated internet users have the smallest and largest values in Africa and Europe, respectively. America has the lowest values of the first quartile, median, third quartile, and maximum values associated with the estimated education index values among the continents.

The summary of hydro-social equations performance is listed in Table 8, where it is seen the best models’, performances are such that *PD* > *PRUP* > *EI* > *IU*, *PD* > *IU* > *EI* > *PRUP*, *PD* > *IU* > *PRUP* > *EI*, *PD* > *PRUP* > *IU* > *EI* and *PD* > *EI* > *IU* > *PRUP* for Africa, America, Asia, Europe, and Oceania, respectively.

**Table 8:** Summary of hydro-social equations performance.

	**Africa**	**America**	**Asia**	**Europe**	**Oceania**
Hydro-social equations	*PD*	*PD*	*PD*	*PD*	*PD*
	*PRUP*	*IU*	*IU*	*PRUP*	*EI*
	*EI*	*EI*	*PRUP*	*IU*	*IU*
	*IU*	*PRUP*	*EI*	*EI*	*PRUP*

**Figure 13:**
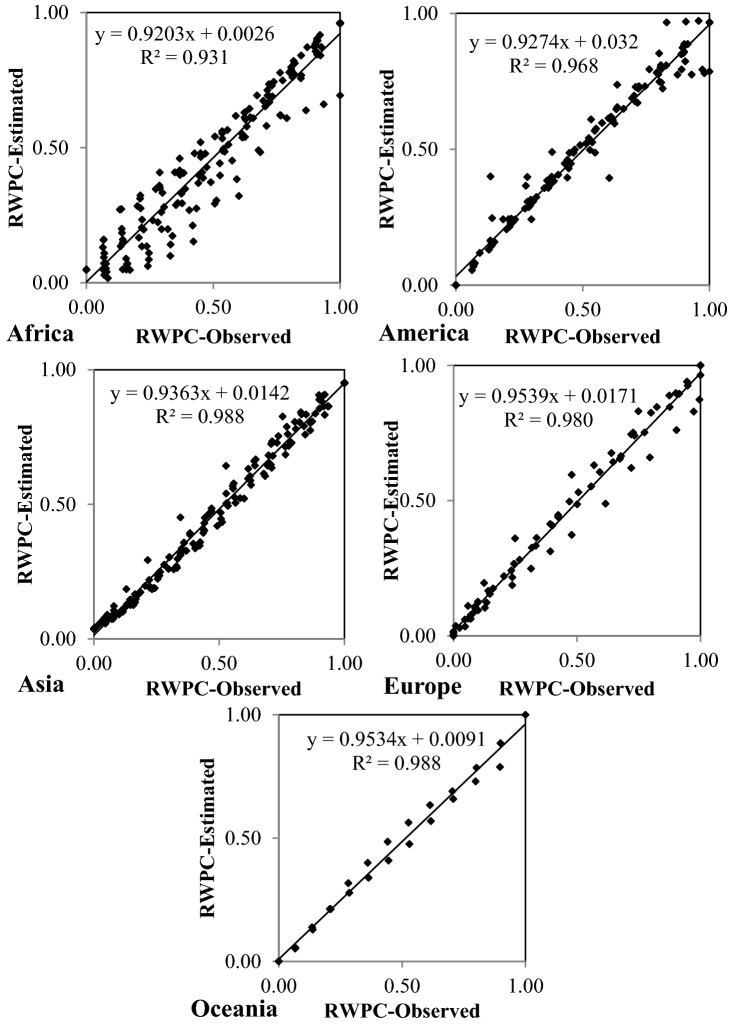
Observed and estimated RWPC parameters during the test period in the world’s continents.

This paper’s results indicate the pattern of association between social parameters and water resources is complex. Renewable water per capita was estimated using social indicators PRUP, PD, IU, and EI based on gene expression programming. The results of GEP to estimate RWPC corresponding to the testing period in the world’s continents as listed in Table 9. The values of RMSE for optimal GEP models equaled 0.089, 0.058, 0.042, 0.049, and 0.036 for Africa, America, Asia, Europe, and Oceania, respectively. Figure 13 displays the observed and estimated RWPC parameter during the test period in the world’s continents. The equations obtained with the optimal models for the renewable water per capita in Africa, America, Asia, Europe, and Oceania are listed in Table 10. The fitted equations can be applied at variable spatial and temporal scales. The derived equations imply that water resources in Africa and Oceania are governed by the PRUP, PD, IU, and EI indicators. Also, the PRUP, PD, and IU indicators in Europe and PD and IU indicators in America and Asia have the most influence on their water resources status. The association between social parameters and water resources in all continents is variable. The linking of these social indicators with the per capita renewable water is a function of the countries' cultural and economic conditions, thus bearing on the future management and policymaking across continents. This study’s results concerning hydro-social indicators are consistent with the findings by Forouzani et al.^2^, Carey et al.^15^, Lima et al.^25^, Pande et al.^7^, Diep et al.^26^, and Diaz et al.^22^.

**Table 9:** The results of GEP estimating RWPC corresponding to the testing period in the world’s continents.

Continent	R	RMSE	MAE
Africa	0.965	0.089	0.065
America	0.984	0.058	0.034
Asia	0.994	0.042	0.034
Europe	0.990	0.049	0.031
Oceania	0.994	0.036	0.025

**Table 10:** Mathematical equations governing hydro-social indicators.

Continent	Equations
Africa	$$\begin{aligned} RWPC_{ts} & = (2IU_{ts} .EI_{ts} )/(EI_{ts} + PRUP_{ts} - 9.90) + PD_{ts} {\text{Cos}} (IU_{ts} + 14.80) \\ & \quad + \left[ {(0.70 - IU_{ts} )^{2} + (EI_{ts} + 0.64PD_{ts} )} \right]^{1/2} \\ \end{aligned}$$
America	$RWPC_{ts} = (PD_{ts} - 1)^{2} - PD_{ts}^{2} + 2PD_{ts} - IU_{ts}$
Asia	$RWPC_{ts} = Exp\left[ {0.27IU_{ts}^{2} - IU_{ts}^{{}} Exp(IU_{ts} )} \right] + 0.03(IU_{ts} - PD_{ts} ) - 0.06$
Europe	$RWPC_{ts} = 3PD_{ts} (PD_{ts} - 0.80)^{2} .{\text{Sin}} (IU_{ts} - PRUP_{ts} )$
Oceania	$$\begin{aligned} RWPC_{ts} & = EI_{ts} IU_{ts} PD_{ts} (IU_{ts}^{1/3} - PD_{ts} ) + {\text{Cos}} (2.06IU_{ts} ) \\ & \quad + PRUP_{ts} \left[ {IU_{ts} - {\text{Cos}} ((EI_{ts} PRUP_{ts} + IU_{ts} )^{1/2} )} \right] \\ \end{aligned}$$

This paper’s results establish the importance of examining the interactions between climate, the status of water resources, and social indicators. The state and social conditions of a country reflect the status of its water resources. Therefore, this study has shown how significant an impact the management and planning of a country can have on its water resources. Each successful water resources project rests on a successful social setting.

## Concluding remarks

One of the most critical issues in the water resources systems is its social context, which poses many challenges in systems analysis. Therefore, the impact of social indicators on water issues, and vice versa, is crucial. On the other hand, direct measurement of social indicators and their evaluation in water resources management is complicated, time-consuming, and expensive. Therefore, it would be beneficial to implement development plans, and management measures better if we can estimate social indicators and water indicators and their interrelationship. Therefore, studies on hydro-social indicators would help us predict social changes related to water resources and vice versa in management plans to face fewer consequences. Social conditions may preclude meeting water-resources planning objectives. Therefore, such planning must be socially grounded for its success. This study assesses several social indicators, i.e., the proportion of rural to urban population (PRUP), population density (PD), internet users (IU), and education index (EI) worldwide, and concludes these indicators have a high correlation with the per capita renewable water. These social indicators must be considered in water policy decisions and planning for sustainable water management and planning. It is concluded the modeling the association of hydro-social indicators with the per capita renewable water using the soft-computing methods is viable and insightful. The performance criteria of the GEP models performed better than those of the ANFIS-SC and ANN-LM models for the world’s continents. This paper shows it is possible to estimate the water status and social indicators of a society based on hydro-social equations developed when there is a paucity of information about the social status. These estimates are useful for water resources management. This study has shown a successful application of hybrid soft computing to determine functional relations between socio-economic parameters and water and environmental resources parameters.

## Recommendations

Some specific recommendations for improving future research are as follows:

- Examining other quantitative and qualitative social indicators.
- Examining other indicators such as environmental, economic, cultural, and political indicators.
- Applying other models of soft computing methods for exploring hydro-social relationships.
- Applying soft computing methods in examining the interrelationships between social indicators and water resources indicators to specific issues of water management, such as flood and drought management.

## Data Availability

All the required data have been presented in our article.
